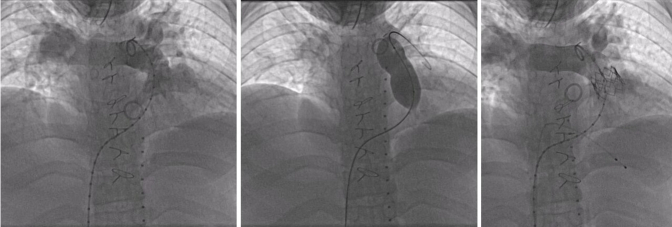# Long-Term Outcomes With Expanded Polytetrafluoroethylene Valved Conduits in Pediatric Patients

**DOI:** 10.1016/j.atssr.2024.04.021

**Published:** 2024-05-10

**Authors:** Yoshio Ootaki, Ashok Muralidaran, Inder Mehta, Michael J. Walsh, Ross M. Ungerleider

**Affiliations:** 1 Oregon Health & Science University, Portland, Oregon; 2 Driscoll Children’s Hospital, Corpus Christi, Texas; 3 Atrium Health Wake Forest Baptist, Winston Salem, North Carolina; 4 Institute for Integrated Life Skills, LLC, Bermuda Run, North Carolina

## Abstract

**Background:**

The expanded polytetrafluoroethylene (ePTFE) valved conduit (VC) has been reported for pulmonary valve replacement (PVR). The purpose of this study was to review long-term outcomes of our trileaflet ePTFE VC.

**Methods:**

This multicenter study was performed with institutional review board approval from each institution. Our VC is fashioned from commercially available ePTFE tube grafts (for the conduit) and 0.1-mm-thick ePTFE membrane (for the trileaflet material). The patients were followed up in our clinic. Valve function was assessed by echocardiography in the operating room and at follow-up clinic visits after implantation.

**Results:**

Fifty-five patients received ePTFE VC between 2012 and 2023 (16-28 mm in diameter). Patients’ age at the time of implantation ranged from 6 months to 20 years (median, 7.5 years). Clinical follow-up ranged from 4 days to 10.1 years (average, 3.6 years). There were no hospital deaths. There were 2 non–valve-related late deaths. There have been no cases of endocarditis. Two patients required balloon dilation for the distal pulmonary artery stenosis, and 1 patient required residual ventricular septal defect closure. Five patients have received transcatheter PVR (TPVR) because of increased pressure gradient across the VC. Freedom from TPVR at 10 years was 90%. No valves required explantation or surgical replacement.

**Conclusions:**

Compared with historical data for other PVR options, our ePTFE VC shows excellent long-term performance and, when required, provides an easily accessible “landing zone” for TPVR. Our technique is easily learned, is reproducible, and can be a valuable option for surgeons performing PVR in pediatric patients.

**In Short**

▪ This study demonstrated excellent long-term durability of expanded polytetrafluoroethylene valved conduit for surgical pulmonary valve replacement (PVR).
▪ Combined approach with expanded polytetrafluoroethylene valved conduit and transcatheter PVR reduces the number of surgeries for patients who require PVR.

Pulmonary valve replacement (PVR) is 1 of the most frequent congenital heart surgical procedures performed on adolescents and young adults. Multiple surgical options, including autologous pericardium, mechanical valves (although these are not commonly used in the pulmonary position), allografts, and bioprosthetic valves (bovine pericardium or porcine aortic valves as well as bovine jugular vein conduits), are available. More recently, transcatheter PVR (TPVR) has been widely available. None of these valves are ideal, and each has problems with durability and freedom from stenosis or insufficiency and can require reintervention, particularly in the pediatric population.

The expanded polytetrafluoroethylene (ePTFE) valved conduit (VC) has been reported for surgical PVR (SPVR) since 2005.^1^ Successful implantation of ePTFE VC has been reported for >1000 patients with different designs.^2^ Our group reported successful midterm follow-up after implantation of simplified trileaflet ePTFE VC in 2018.^3^ Our longest follow-up to date has exceeded >10 years. The purpose of this study was to review the long-term outcomes of our ePTFE VC.

## Patients and Methods

### Patients

The inclusion criterion was the use of handmade trileaflet ePTFE VC for correction of the pulmonary valve. All other techniques, including bicuspid and monocusp valves, were excluded. This research study was approved by the institutional review board in each institute.

### Construction of the Conduit

**Supplementary Figure 1:**
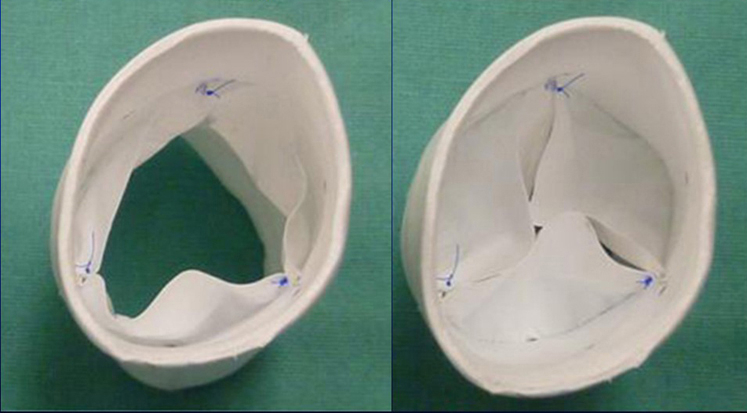


We construct this VC (Video) with commercially available PTFE conduits and 0.1-mm-thick PTFE membrane for the leaflet material (W. L. Gore & Associates). We created 28-mm conduits by adding a piece of graft from a 24-mm graft. As the patient is being prepared for surgery by the anesthesia team, the surgeon creates the conduit on a sterile back table in the operating room. A trileaflet VC was created with our technique that has previously been described (Supplemental Figure 1).^3^ We currently use CV-5 PTFE sutures (W. L. Gore & Associates) for leaflet reconstruction.

### Patient Follow-up

Function of the implanted ePTFE VC was evaluated in the operating room by intraoperative echocardiography as well as during routine clinical outpatient follow-ups by clinical assessment and echocardiography. If there were any concerns of significant valve failures or estimated right ventricular pressure measurements that exceeded two-thirds systemic pressure, the patients were sent to cardiac catheterization to assess heart and valve functions.

### Statistical Analysis

Univariate analysis for survival, freedom from reintervention, freedom from TPVR, and freedom from SPVR were performed by the Kaplan-Meier method. All analysis was carried out with Microsoft Excel.

## Results

**Figure 1:**
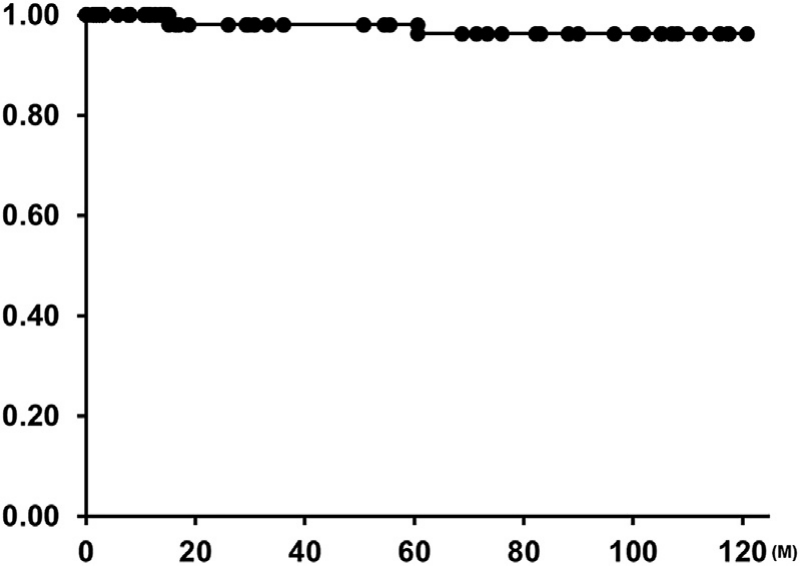
Kaplan-Meier curve of survival.

From June 2012 to August 2023, we implanted a trileaflet ePTFE VC in 55 patients in 3 study institutes (Wake Forest School of Medicine, Driscoll Children’s Hospital, and Oregon Health & Science University). The median age at the time of implantation was 7.5 years (range, 0.5-20.3 years). The median body weight was 23.0 kg (range, 6.7-140.2 kg). The demographic data are described in Supplemental Table 1. Of the 55 patients in our cohort, there were no hospital deaths after implantation. Mean follow-up was 3.6 years (1 month–10.1 years). One patient had mediastinitis after SPVR, which was medically treated without VC explantation. Valve function was excellent on 4-year follow-up echocardiography in this patient. No patient had infectious endocarditis during our follow-up. There were 2 non–valve-related late deaths. One patient, who had a Ross operation and implantation of an ePTFE VC in the pulmonary position with an uneventful hospital course, died 15 months after the surgery of unknown reason. This patient showed no regurgitation and minimal pressure gradient (peak of 20 mm Hg) in the ePTFE VC at 14 months of follow-up. One patient who had implantation of an ePTFE VC in the pulmonary position with an uneventful hospital course died 48 months after the surgery as a result of a traffic accident. His most recent echocardiogram before this accident showed no significant valve failure. Overall patient survival was 96% at 10 years (Figure 1).

**Figure 2:**
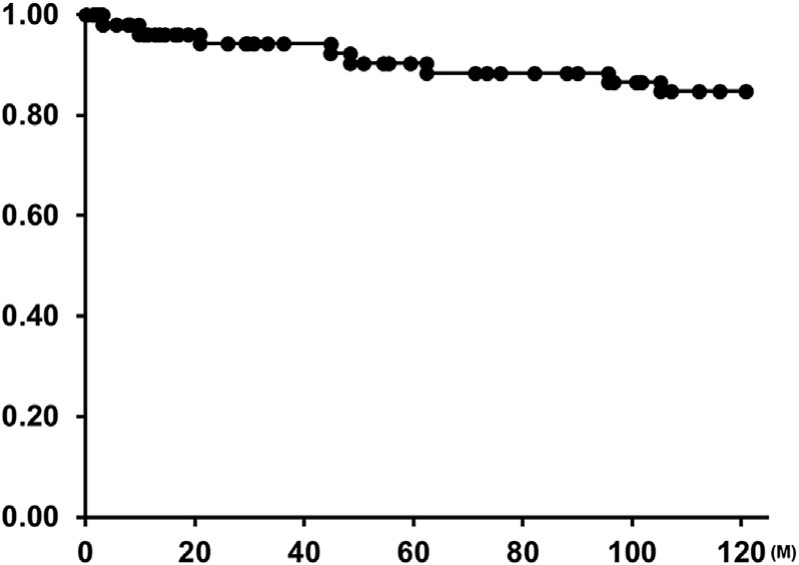
Kaplan-Meier curve of freedom from reintervention.

**Figure 3:**
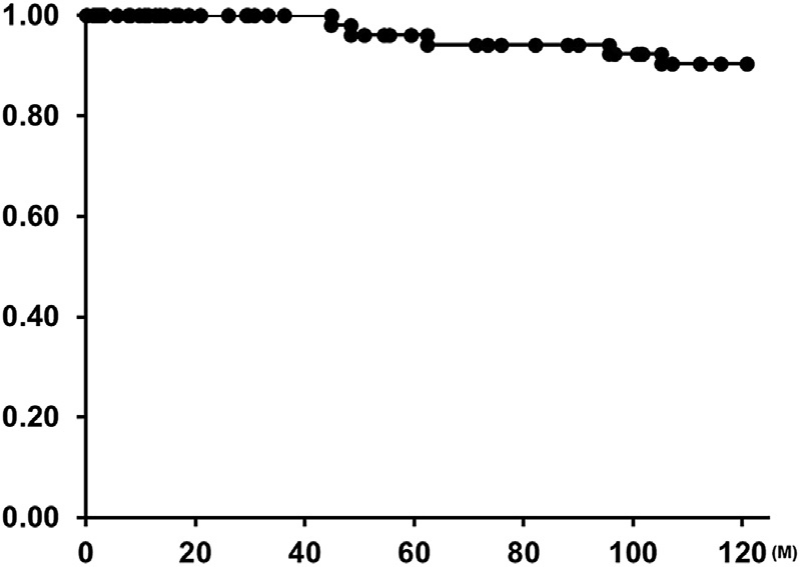
Kaplan-Meier curve of freedom from transcatheter pulmonary valve replacement.

**Figure 4:**
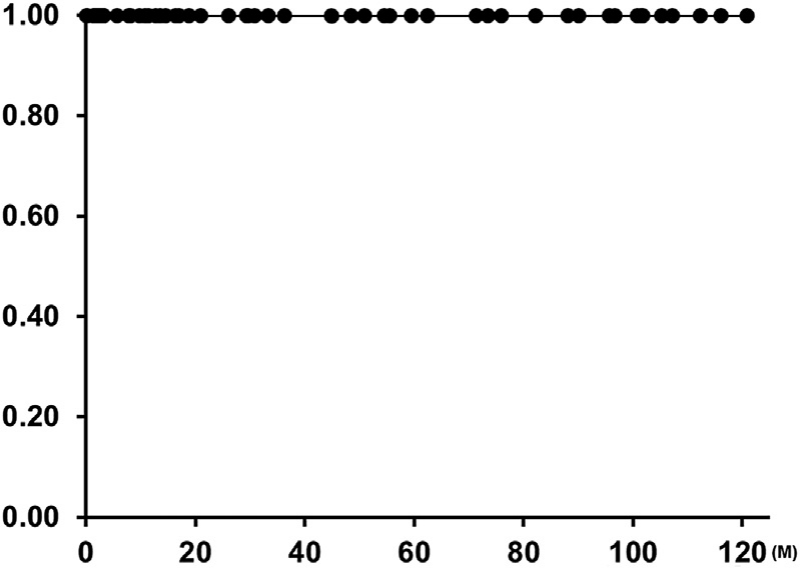
Kaplan-Meier curve of freedom from surgical pulmonary valve replacement.

There were 8 reinterventions during the follow-up period. Two of these were patients early in our series who had balloon pulmonary arterioplasty for stenosis at the distal anastomosis. One patient required residual ventricular septal defect closure after 3 months, although ePTFE valve function was normal. Five patients have received TPVR with Melody valve (Medtronic; n = 4) or Sapien valves (Edwards Lifesciences; n = 1) because of increased pressure gradient across the VC (20 mm in 1 patient and 24 mm in 4 patients). The freedom from reintervention was 85% at 10 years (Figure 2), and the freedom from TPVR was 90% at 10 years (Figure 3). No ePTFE VC required explantation or SPVR (Figure 4). ePTFE valve function demonstrated less than moderate insufficiency in 89.5% of patients (43/48).

## Comment

Implantation of ePTFE VC was first described by a Japanese group in 2002.^4^ There have been several different techniques to create ePTFE VC since then. Quintessenza and associates^1^ reported creation of bicuspid fan-shaped ePTFE VC. Miyazaki and associates^4^ reported the technique for creating a trileaflet fan-shaped ePTFE VC with bulging sinuses. This technique requires a heating and resterilization process to create sinuses in PTFE conduits and is less convenient in situations in which the valve needs to be created at the time of SPVR. A Korean group^5^ reported use of a rectangular trileaflet ePTFE VC. Many of the Japanese and Korean techniques require a resterilized process after the creation of ePTFE VC. We reported a simple technique for creating a trileaflet fan-shaped ePTFE VC without the need for heating or resterilization.^3^ One of the advantages of trileaflet VC over bicuspid leaflet is that a “shorter” VC can be obtained, which can provide a substantial advantage during SPVR. Similar simple techniques with trileaflet ePTFE VC were reported from China^6^ and Taiwan^7^ (Supplemental Table 2). It is difficult to compare long-term results between these different kinds of ePTFE VC. However, as longer term follow-up data are becoming available, it is evident that the durability of these unique VCs is reproducible, especially in larger VCs, which are unlikely to be outgrown.^8^

Prosthetic pulmonary valve endocarditis has been a serious complication after TPVR or SPVR. Malekzadeh-Milani and associates^9^ reported that person-time incidence rates of infectious endocarditis were 2.4 cases per 100 person-years after TPVR or SPVR. There have been reports that Contegra pulmonary valved conduit (Medtronic) and Melody transcatheter valves have higher incidence of infectious endocarditis. However, the incidence of infectious endocarditis after implantation of ePTFE VC has been reported to be low.^1^^,^^3^, ^4^, ^5^, ^6^, ^7^ In our patients after ePTFE VC implantation, there have been no episodes of infectious endocarditis during the follow-up period. Fujita and associates^8^ reported that person-time incidence rates of infectious endocarditis were 0.13 cases per 100 person-years after PVR with ePTFE VC. This lower risk for having infectious endocarditis after ePTFE VC implantation is another advantage compared with other available prosthetic pulmonary valve materials.

TPVR is a minimally invasive approach for pulmonary stenosis or insufficiency for both native pulmonary valves and failed prosthetic pulmonary valves. One of the challenges of TPVR is compression of coronary arteries after placement of the TPVR. A routine procedure to evaluate for the risk of this complication has been prestenting balloon dilation and coronary angiography. Despite this precaution, there is still a risk of coronary artery compression after deployment of the transcatheter valve. Another challenge of TPVR is rupture of the native pulmonary artery or previously placed conduit after balloon dilation. Whereas covered stents can be deployed as a rescue procedure in the situation of pulmonary conduit rupture, hemodynamic instability and massive transfusion are potential consequences of covered stent deployment.

TPVR inside the ePTFE VC is theoretically ideal because the ePTFE tube has smooth and equal internal diameter that creates a “landing zone” for a transcatheter valve. We have 5 patients who received TPVR after ePTFE VC SPVR, and none of these cases had difficulty in placement of the TPVR (Supplemental Figure 2). Even after deterioration of ePTFE valve function, typically calcified ePTFE valve leaflets can be easily dilated with stent dilation. Freedom from explantation or SPVR in our patients was 100% in 10 years. Khanna and associates^10^ reported that a redo sternotomy for SPVR past the third reoperation increased mortality and morbidity. Reducing the number of surgeries with a combination of ePTFE VC and eventual (if needed) TPVR will likely contribute to reducing mortality and morbidity for these patients.

Another possible advantage of this ePTFE VC is its cost. Excluding any additional surgical fees for creation of the VC, a 24-mm ePTFE VC costs $1171 for materials, including sutures used for leaflet construction. In the United States, pulmonary valve allograft (Artivion), Hancock valved conduit (Medtronic), and Epic plus supra aortic valve (Abbott Laboratories) cost $25,000, $6500, and $6000, respectively. Whereas medical expenses increase each year, Medicare and Medicaid services do not fully reimburse for many of these costs. In this era, as we strive toward adding value in this increasingly common operation, the improved long-term outcomes and reduced cost of ePTFE VC are appealing.

### Limitations

There are obviously several limitations in this study. This is not a randomized or control-matched study to assess various types of pulmonary valve material, and we could not directly compare our ePTFE VC with other types of valves. Our population of patients is relatively older, and we have not implanted ePTFE VC sizes below 16 mm in diameter. There is also selection bias because the wide availability of Melody and Sapien TPVR often leads to the recommendation for this option in lieu of SPVR with ePTFE VC. A future study might be able to compare the outcomes of patients receiving TPVR with those of patients receiving surgical ePTFE VC at our multiple institutions, trying as much as possible to compare patients who were likely candidates for either procedure. Another possible limitation is that there is no guideline in terms of anticoagulation for an ePTFE VC. At this moment, we recommend daily aspirin for the patients who received ePTFE VC in the pulmonary position according to the American College of Cardiology/American Heart Association guideline. Postoperative management should be standardized after implantation of these ePTFE VCs. Finally, ideal timing of TPVR is unknown once ePTFE valve function deteriorates. We proceeded with TPVR when right ventricular pressure exceeded more than two-thirds systemic pressure and if the gradient was related to the valve and not amenable to balloon dilation at a suture line. Continuous follow-up will be necessary for this innovative ePTFE VC.

### Conclusion

Compared with historical data for other PVR options, our ePTFE VC shows excellent long-term performance and, when required, provides an easily accessible landing zone for TPVR. There was no SPVR in our patients, no endocarditis, and no valve-related deaths. Our technique is easily learned, is reproducible, and can be a valuable option for surgeons performing PVR in pediatric patients.

## Figures and Tables

**Supplementary Figure 2:**