# Supplementary material for: Long-Term Outcomes With Expanded Polytetrafluoroethylene Valved Conduits in Pediatric Patients
Source: Ann Thorac Surg Short Rep. 2024 May 10;2(4):810–4. doi: 10.1016/j.atssr.2024.04.021 (PMC11708714; doi:10.1016/j.atssr.2024.04.021)
Supplement: Supplementary Table 1 [file mmc1.docx]

Supplemental Table 1. Demographic data.

| Demographic characteristics |  |
| --- | --- |
| Median age at operation | 7.5 years |
| Median body weight | 23.0 kg |
| Sex (male/female) | 33/22 |
| Clinical diagnosis / procedure | N (%) |
| s/p Tetralogy of Fallot repair, severe PI | 19 (34.5) |
| RV-PA conduit stenosis | 15 (27.3) |
| Aortic valve disease requiring Ross procedure | 10 (18.2) |
| Rastelli operation | 4 (7.3) |
| Pulmonary stenosis / insufficiency | 4 (7.3) |
| Tetralogy of Fallot with absent pulmonary valve | 3 (5.4) |
| Graft sizes | N (%) |
| 16 mm | 2 (3.6) |
| 18 mm | 5 (9.1) |
| 20 mm | 15 (27.3) |
| 24 mm | 32 (58.2) |
| 28 mm | 1 (1.8) |

PI: pulmonary insufficiency, RV-PA: right ventricle to pulmonary artery
