# Supplementary material for: Long-Term Outcomes With Expanded Polytetrafluoroethylene Valved Conduits in Pediatric Patients
Source: Ann Thorac Surg Short Rep. 2024 May 10;2(4):810–4. doi: 10.1016/j.atssr.2024.04.021 (PMC11708714; doi:10.1016/j.atssr.2024.04.021)
Supplement: Supplementary Table 2 [file mmc2.docx]

Supplemental Table 2. Type of ePTFE VC.

| **year** | **author** | **# of leaflets** | **shape** | **sinus** | **re-sterilization** | **ref** |
| --- | --- | --- | --- | --- | --- | --- |
| 2005 | Quintessenza | 2 | fan-shape | No | No | 1 |
| 2011 | Miyazaki | 3 | fan-shape | Yes | Yes | 4 |
| 2018 | Ootaki | 3 | fan-shape | No | No | 3 |
| 2019 | Choi | 3 | rectangle | No | Yes | 5 |
| 2022 | Shi | 3 | fan-shape | No | No | 6 |
| 2023 | Chang | 3 | fan-shape | No | No | 7 |
