# Supplementary material for: Long-Term Outcomes With Expanded Polytetrafluoroethylene Valved Conduits in Pediatric Patients
Source: Ann Thorac Surg Short Rep. 2024 May 10;2(4):810–4. doi: 10.1016/j.atssr.2024.04.021 (PMC11708714; doi:10.1016/j.atssr.2024.04.021)
Supplement: Supplementary Figures Legend [file mmc3.docx]

**Supplemental Figure legends**

**Supplemental Figure 1**: Trileaflet ePTFE valved conduit in open (left) and closed (right) position.

**Supplemental Figure 2**: Placement of Sapien valve inside the ePTFE valved conduit. Pre-implantation (left), balloon dilatation inside the ePTFE valved conduit (middle), and post-implantation (right).
